# Post-Flowering Photoperiod Sensitivity of Soybean in Pod-Setting Responses

**DOI:** 10.3390/biology13110868

**Published:** 2024-10-25

**Authors:** Zhihui Sun, Limei Yuan, Yulin Wang, Ran Fang, Xiaoya Lin, Haiyang Li, Liyu Chen, Yichun Wu, Xin Huang, Fanjiang Kong, Baohui Liu, Sijia Lu, Lingping Kong

**Affiliations:** 1 Guangdong Key Laboratory of Plant Adaptation and Molecular Design, School of Life Sciences, Guangzhou University, Guangzhou 510006, China; 2 Innovative Center of Molecular Genetics and Evolution, School of Life Sciences, Guangzhou University, Guangzhou 510006, China

**Keywords:** flower abscission, photoperiod-sensitive, post-flowering, pod setting, style morphology, RBOH, soybean

## Abstract

The development of soybean (*Glycine max*) is regulated by the photoperiod, with genes related to photoperiod sensitivity primarily focused on the flowering time. However, their roles in post-flowering reproductive development and the mechanisms by which the photoperiod affects them are not yet determined. In this study, we found that pod formation is sensitive to the photoperiod. Long-day conditions tended to extend the time from flowering to pod formation, and the first wave of flowers tended to fall off. Additionally, the photoperiod affected the pistil morphology; under short-day conditions, the stigma had a curved hook-like structure that facilitated better interaction with the filaments when pollen was released, ultimately influencing the timing of pod formation. Photoperiod-insensitive mutants, lacking *E1* family and *Evening Complex* (EC) genes, showed no difference in the pod formation time under long-day or short-day conditions. Hormone content analysis and transcriptome data analysis indicated that various hormones, reactive oxygen species (ROS) burst pathway signals, and the application of sucrose solution in vitro might influence floral organ abscission.

## Simple Summary

Plants measure day length (photoperiod) to regulate seasonal growth and flowering. Photoperiodic flowering has been well studied, but less is known about how the photoperiod affects reproductive growth after flowering. In this study, we investigated the effects of the photoperiod on pod formation during soybean (*Glycine max*) development. Compared to short-day conditions, long-day conditions extended the time from flowering to pod formation and led to the abscission of the first wave of flowers. However, transferring plants from long-day to short-day conditions can reverse the phenotypes of delayed pod formation and flower abscission caused by long days. Hormone levels and transcriptome data indicated that hormones, reactive oxygen species signaling pathways, and sucrose application all influenced flower organ abscission. This research provides new insights into how the photoperiod regulates reproductive development in soybeans, which has significant social value for improving agricultural production efficiency and optimizing soybean cultivation.

## 1. Introduction

The photoperiod is a rhythmic change in the amount of light received by an organism [1,2]. Plants sense the photoperiod, which enables them to adjust their flowering time according to seasonal changes in light to adapt to growing conditions at different latitudes [3,4,5]. In addition to regulating the flowering time, the photoperiod also affects physiological processes, such as photosynthesis, growth rhythm, and nutrient metabolism of plants. Plants adjust the intensity and time of photosynthesis by sensing the photoperiod to maximize the use of light energy for nutrient synthesis and growth development. The photoperiod is also closely related to processes such as the distribution of photosynthetic products, carbon metabolism, and the synthesis of phytohormones, directly affecting the growth rate and morphological structure of plants.

Soybean is a typical short-day (SD) crop, which is very sensitive to changes in the photoperiod. Usually, one variety or germplasm resource is suitable for planting in a particular narrow latitude range because modern cultivated soybean varieties require such specific photoperiods [6,7]. The wide genomic adaptability of soybean is mainly achieved through changes in the multiple genes or quantitative trait loci that control the flowering and reproductive period. A growing number of photoperiod-responsive gene loci have been identified and analyzed at the molecular level, including the *E* series *E1*–*E4* [8,9,10,11] and *E9* [12,13,14], *Time of flowering* (*Tof5*) [15], *Tof11*, *Tof12* [16], and *Tof16* [17], *LUX ARRHYTHMO* (*Lux*) [5], and *J* [*7*]. The flowering time loci *E1*, *E2*, *E3*, *E4*, *Tof11*, and *Tof12* play a role in regulating long-day (LD) insensitivity (where mutants of these genes tend to flower earlier, even under non-inductive LD conditions, such as high latitudes) [16,18]. Over 80% of low-latitude soybean varieties harbor different mutant alleles in the *J* and *Tof16* genes, suggesting that *Tof16* and *J* play a significant role in soybean adaptation to SD photoperiods (mutants of these genes tend to have long juvenile periods and flower late under induced SD (low latitude) conditions) [17].

The photoperiodic response of soybeans not only operates during the pre-flowering growth stages but also plays a crucial role in post-flowering vegetative [18,19] and reproductive growth processes [20,21]. During the post-flowering stages, plants remain sensitive to the photoperiod, and this sensitivity is also regulated by maturity genes [22,23]. The interaction between genes and the environment that control the reproductive period directly affects various phenotypic characteristics in the post-flowering stages, such as pod setting [24], pod development [20,25,26], terminal vegetative growth, and reproductive growth [18,27,28]. The extended duration of R3–R6 under longer photoperiods tends to increase pod and seed numbers [20]. The post-flowering photoperiod extension delays individual fruit development in soybean from the R1 stage to the seed-filling stage [24]. However, while we have observed the influence of the photoperiod on the flowering to pod-setting process, the molecular mechanisms involved remain unclear. Apart from maturity genes, genes potentially involved in regulating the flowering to pod-setting process may include those related to light signal transduction, plant hormone regulation, carbon metabolism, and nutrient transport. These genes interact through complex signaling networks, regulating soybean growth, development, and yield formation during the post-flowering stages. In-depth studies of these genes can help us comprehensively understand the growth regulatory mechanisms of soybeans, providing a scientific basis for improving soybean yield and quality.

Long days lengthen the flowering period and thereby increase the number of opened flowers on lateral racemes. During the post-flowering phase, seed-filling effectiveness is delayed on primary racemes (dominant positions), enhancing the pod number on lateral racemes (usually dominated positions) at some main stem nodes under long-day conditions [24]. This phenomenon is often observed under artificial light conditions in greenhouses or growth chambers: under long-day conditions (e.g., 16 h light/8 h dark), the first flowers to bloom of most soybean varieties gradually fall off instead of developing into pods. In contrast, under artificial short-day conditions (e.g., 12 h light/12 h dark), flowers begin to produce pods more quickly. Prolonged daylight hours also delay the time for soybean flowers to develop into pods, extending the pod initiation period without altering the rate of pod elongation [24]. This indicates the influence of the photoperiod on the pod development process, while also suggesting the potential involvement of other factors affecting pod development and maturation. There is a complex relationship between pod abscission and photoperiodic responses. Environmental stresses, such as low-light radiation conditions, are important factors that may induce flower buds’ abscission [29]. Studies in different species have shown that flower/fruit abortion is determined by the availability of assimilates [30,31]. When seeds enter the linear phase of growth and accumulate assimilates at their maximum rate, they become a relatively large reproductive sink that may limit upcoming flowering, resulting in flower abortion to allow the older organs to finish their development [32]. Sugar signaling plays a potential central role in regulating lotus (*Nelumbo nucifera*) flower bud abortion; for example, the overexpression of *Trehalose-6-P Synthase 1* (*TPS1*) in lotus significantly decreased the flower bud abortion rates in both normal-light and low-light environments [29]. This illustrates the importance of sugar signals in regulating post-anthesis development, possibly affecting soybean pod development and maturation by regulating the distribution and utilization of assimilates. It is proposed that flower abortion could be mediated by hormonal induction, potentially by the candidate hormone indole-3-acetic acid (IAA) [33]. Abscisic acid could also be involved because it has an inhibitory role on flowering [34].

Flower and pod abscission are important factors affecting soybean crop yields. Therefore, analyzing the physiological mechanisms of photoperiodic regulation on flowering and subsequent pod development is of significant importance for promoting crop breeding and genetic improvement. In this study, we observed that the time from flowering to pod formation on the whole soybean plant was longer under LD conditions than under SD conditions. Such differences under different photoperiods were not observed in photoperiod-insensitive soybean genotypes, indicating that the period between flowering and pod setting was sensitive to day length. Furthermore, we found the pod-setting signal was mainly induced and transmitted by leaves. We, therefore, showed that the photoperiod affects the various stages of soybean growth and development. Further research into the molecular mechanisms regulating the time between flowering and pod setting will be helpful for improving soybean yields through the reduction of flower and pod abortion.

## 2. Materials and Methods

### 2.1. Plant Materials, Growth Conditions, and Phenotyping

The soybean cultivars Williams 82 (W82; *e1-as/E2/E3/E4*) [35] and Harosoy (*e1-as/e2/E3/E4*) [11] were used in this study. W82 is more sensitive to long photoperiods than Harosoy. Using W82 as the wild-type, homozygous transgenic *lux* double mutants (*lux-2m*, *lux1*, and *lux2-2*, as published [5]), and *e1* triple mutants (*e1-3m* and *e1/e1la/e1lb* mutant type, as described [36]), the wild-type plants were used for the experiments. Plants were grown under artificial SD (12 h light/12 h dark), artificial LD (16 h light/8 h dark), and ultra-long-day (20 h light/4 h dark) conditions in a greenhouse or a growth chamber, with a light intensity of 240 μmol m^–2^ s^–1^ and a temperature of 25 °C. According to the description of the developmental stages of soybean [37], the reproductive stages R1 and R2 were based on flowering, R3 and R4 on pod development, R5 and R6 on seed development, and R7 and R8 on maturation. Flowering time was recorded at the R1 stage as the number of days from seedling emergence to the first open flower at any node on the main stem. The pod-setting time was recorded when any node at the four upmost produced a pod with a length of 0.5 cm. At least five plants were detected for each line.

### 2.2. Transfer Between Different Photoperiod Conditions

W82 plants were grown under LD (16 h light/8 h dark) conditions until R1 in the greenhouse, after which half were transferred into SD (12 h light/12 h dark) conditions (named LD_SD group), with the others remaining in the LD (16 h light/8 h dark) treatment (named continuous LD group or LD_LD group). Pod-setting times were then measured after the transferred treatments. The duration of these treatment was 60 days.

### 2.3. Branch-Specific Photoperiod Treatments

W82 plants were grown under LD (16 h light/8 h dark) conditions until the fifth day after emergence in the growth chamber. To ensure branching in each plant, the shoot apical meristems (SAMs) were cut to remove the apical dominance and promote the development of lateral branches. All plants were grown in LD conditions until reaching the R1 stage, after which the branches were subjected to treatments of different photoperiodic combinations. In one set of experiments, the light phase of one branch was shortened to 12 h using black bags to exclude light. The bags were removed each day and then replaced at Zeitgeber time 12 (ZT 12). When the light was on (at ZT 0), they were removed. To remove any phenotypic differences caused by this bagging, another branch was covered with transparent plastic bags as the LD control. In another set of experiments, both branches were covered with transparent plastic bags and subjected to LD conditions. To further demonstrate the role of leaves in perceiving the photoperiod and controlling the pod initiation time, all leaves of branches under different photoperiod conditions were removed, with another branch retaining its leaves as the control.

### 2.4. Pollen Germination Analysis

The pollen germination experiments were based on in vitro and in vivo pollen germination. In brief, for in vitro pollen germination, mature pollen grains of W82 under LD and SD conditions were dispersed on pollen germination medium containing 10% sucrose, 0.01% boric acid, 5 mM CaCl_2_, 5 mM KCl, 1 mM MgSO_4_, pH 7.5, and 1.5% agar [38]. Germination mediums were then incubated at 25 °C for 7 h. Pollen germination was observed under a Carl-Zeiss Axio Imager A2 microscope (Carl-Zeiss, Jena, Germany). Pollen tube lengths were measured by Image J software (Version 1.8.0). For in vivo germination experiments, pollen grains were applied on stigmata of W82 under LD and SD conditions. After 20 h, the hand-pollinated pistils were fixed in a solution of 45%:6%:5% acetic acid/ethanol/formaldehyde for 2 h, washed with 70% ethanol, 50% ethanol, 30% ethanol, and ddH_2_O for 10 min each, and then treated with 8 M NaOH overnight. Samples were washed three times with ddH_2_O and stained with aniline blue solution (0.1% aniline blue, 108 mM K_3_PO_4_) for more than 2 h [39]. Stained samples were observed under a fluorescence microscope (Zeiss Axio Imager A2).

### 2.5. Transcriptome Analysis

Flower bud samples before flowering were collected at Zeitgeber time 4 at the R1 stage under LD (16 h light/8 h dark) and SD (12 h light/12 h dark) conditions of W82, with each sample collected from 5 individual plants. Analysis was conducted on three biological replicate samples. Pistils were detached from the pod. Experimental methods for total RNA extraction, Illumina sequencing, and RNA differential expression analysis were performed following procedures described in previous publications [5]. Genes/transcripts with false discovery rate (FDR) values below 0.05 and absolute fold change ≥ 2 were considered as differentially expressed genes/transcripts. Soybean reference genomes were used in this study, including https://www.ncbi.nlm.nih.gov/datasets/genome/GCF_000004515.6/ (accessed on 1 October 2024) and https://phytozome-next.jgi.doe.gov/info/Gmax_W82_a4_v1 (accessed on 1 October 2024).

### 2.6. Quantitative Reverse-Transcriptase (RT)-PCR

Total RNA was extracted from pistils of flower buds before opening at R1 stage and 1 day, 5 days, and 10 days after the R1 stage in LD_LD and LD_SD groups using TRIzol regent (Invitrogen, Carlsbad, CA, USA). Total RNA was reverse transcribed to cDNA with the M-MLV reverse transcriptase kit (Takara, Kusatsu, Japan). LightCycler 480 SYBR Green I Master (Roche, Indianapolis, IN, USA) was used for quantitative RT-PCR (qRT-PCR) on a Roche LightCycler 480 system (Roche). *Tubulin* was used as an internal control gene. Three biological replications were performed in each test. Primers are listed in Supplementary Table S4.

### 2.7. Phytohormones’ Detection

#### 2.7.1. Chemicals and Reagents

HPLC-grade acetonitrile (ACN) and methanol (MeOH) were purchased from Merck (Darmstadt, Germany). MilliQ water (Millipore, Bradford, PA, USA) was used in all experiments. All of the standards were purchased from Olchemim Ltd. (Olomouc, Czech Republic) and isoReag (Shanghai, China). Acetic acid and formic acid were bought from Sigma-Aldrich (St. Louis, MO, USA). The stock solutions of standards were prepared at the concentration of 1 mg/mL in MeOH. All stock solutions were stored at −20 °C. The stock solutions were diluted with MeOH to working solutions before analysis.

#### 2.7.2. Sample Preparation and Extraction

Fresh flower buds of soybean plants at the R1 stage, cultivated under LD (16 h light/8 h dark) and SD (16 h light/8 h dark) conditions, were harvested for eight classes of plant hormones’ analysis, including auxin, cytokinins (CKs), abscisic acid (ABA), jasmonates (Jas), salicylic acid (SA), gibberellins (GAs), ethylene (the immediate precursor of ethylene, l-aminocyclopropane-l-carboxylic acid (ACC)), and strigolactones (SL).

Samples were promptly frozen in liquid nitrogen and subsequently ground into a fine powder at 30 Hz for 1 min. A portion of 50 mg was accurately weighed into a 2 mL plastic microtube, re-frozen in liquid nitrogen, and then dissolved in a mixture of methanol, water, and formic acid (15:4:1, v/v/v). An internal standard solution (10 μL) at a concentration of 100 ng/mL was added to the extract for quantification purposes. The resulting mixture was vortexed for 10 min and then subjected to centrifugation at 12,000 rpm for 5 min at 4 °C. The supernatant was carefully transferred to clean plastic microtubes, followed by evaporation to dryness. The residue was then reconstituted in 100 μL of an 80% methanol solution (v/v) and filtered through a membrane filter with a pore size of 0.22 μm prior to LC-MS/MS analysis [40,41].

#### 2.7.3. UPLC Conditions

The sample extracts were analyzed using an UPLC-ESI-MS/MS system (UPLC ExionLC™AD (Sciex, Framingham, MA, USA); MS Applied Biosystems 6500 Triple Quadrupole (Sciex), https://sciex.com.cn/ (accessed on 1 October 2024)). The analytical conditions were as follows: LC column—Waters ACQUITY UPLC HSS T3 C18 (Waters, Milford, MA, USA. 100 mm × 2.1 mm i.d., 1.8 µm); solvent system—water with 0.04% acetic acid (A) and acetonitrile with 0.04% acetic acid (B); gradient program—initiated at 5% B (0–1 min), increased to 95% B (1–8 min), maintained at 95% B (8–9 min), and finally returned to 5% B (9.1–12 min); flow rate—set at 0.35 mL/min; temperature—maintained at 40 °C; injection volume—specified as 2 μL [42,43].

#### 2.7.4. ESI-MS/MS Conditions

Linear ion trap (LIT) and triple-quadrupole (QQQ) scans were acquired on a triple-quadrupole linear ion trap mass spectrometer (QTRAP; QTRAP^®^ 6500+ LC-MS/MS System, https://sciex.com.cn/), equipped with an ESI Turbo Ion Spray interface, operating in both positive- and negative-ion mode and controlled by Analyst 1.6.3 software (Sciex). The ESI source operation parameters were as follows: ion source, ESI+/−; source temperature, 550 °C; ion spray voltage (IS), 5500 V (positive) and −4500 V (negative); curtain gas (CUR), 35 psi. Phytohormones were analyzed using scheduled multiple-reaction monitoring (MRM). Data acquisitions were performed using Analyst 1.6.3 software (Sciex). Multiquant 3.0.3 software (Sciex) was used to quantify all metabolites. Mass spectrometer parameters, including the de-clustering potentials (DP) and collision energies (CE) for individual MRM transitions, were performed with further DP and CE optimization. A specific set of MRM transitions were monitored for each period according to the metabolites eluted within this period [44,45].

#### 2.7.5. Detection of Phytohormones

Phytohormones’ contents were detected by MetWare (http://www.metware.cn/ accessed on 1 October 2024) based on the AB Sciex QTRAP6500 LC-MS/MS platform.

### 2.8. Sucrose Solution Spray After R1 Stage

W82 plants grown under LD conditions were sprayed with 50 mg/mL of sucrose solution on their leaves at the R1 stage for 20 days. The blank control group sprayed water without added sucrose, and the pod initiation stage (R3) of the two treatment groups was observed.

### 2.9. Pathway Enrichment Analysis

Pathway-based analysis helps to further understand genes’ biological functions. Kyoto Encyclopedia of Genes and Genomes (KEGG) [46] is the major public pathway-related database [47]. Pathway enrichment analysis identified significantly enriched metabolic pathways or signal transduction pathways in differently expressed genes (DEGs), compared with the whole-genome background. The calculating formula of the *p*-value is:

$$P=1-\sum_{i=0}^{m-1}\frac{\left(\begin{array}{c} M\\ i \end{array}\right)\left(\begin{array}{c} N-M\\ n-i \end{array}\right)}{\left(\begin{array}{c} N\\ n \end{array}\right)}$$

Here, *N* is the number of all genes with a KEGG annotation, n is the number of DEGs in *N*, *M* is the number of all genes annotated to specific pathways, and m is the number of DEGs in *M*. The calculated *p*-value went through FDR correction, taking FDR ≤ 0.05 as a threshold. Pathways meeting this condition were defined as significantly enriched pathways in DEGs.

## 3. Results

### 3.1. Photoperiod Affects the Initiation of Pod-Setting After Flowering

**Figure 1:**
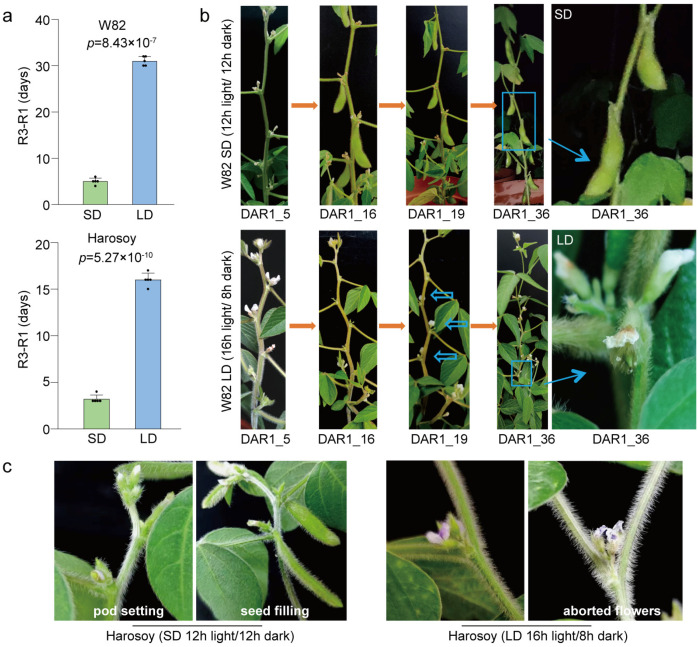
Photoperiod affects the R3 stage of different soybean cultivars. (**a**) The initiation time of pod setting after flowering (duration of R1 to R3 stage) of W82 and Harosoy under short-day (SD; 12 h light/12 h dark) and long-day (LD; 16 h light/8 h dark) photoperiod conditions. (**b**) Phenotypes of flowers and pods of W82 at 5, 16, 19, and 36 days after flowering under SD and LD conditions, respectively (orange arrows present the growth progress). Under the LD condition, the first-opened flowers of W82 gradually fell off instead of developing into pods (DAR1 = 16). New buds were then produced at each node (DAR1 = 19), and the second wave of opened flowers (blue hollow arrows) gradually turned into pods. At about 30 DAR1, the pod-setting stage had just begun under long-day conditions, while the pods had reached the seed-filling stage under the short-day conditions (blue dashed box and blue arrows). Under SD, most of the flowers, including many of the first-opened flowers, successfully initiated pod setting, at about five days after flowering (DAR1 = 5). (**c**) Phenotypes of flowers and pods of Harosoy at DAR1 = 3 and DAR1 = 8. All data are presented as means ± S.E.M. (*n* = 5 plants). One-tailed, two-sample *t*-tests were used to generate the *p*-values. DAR1, days after R1. The bar in the picture represents 0.5 cm.

Under artificial SD (12 h light/12 h dark) and LD (16 h light/8 h dark) conditions, we investigated the flowering time (R1) and the initiation time of podding (R3) of the two cultivars, W82 and Harosoy. The time interval between flowering and pod-setting initiation (R3-R1) varied among different varieties (Figure 1a,b). Under LD conditions, successful pod setting typically took approximately 15–30 days after R1 (approximately 15 days for Harosoy and approximately 30 days for W82; Figure 1a,b). Comparing the time to pod formation under LD and SD conditions, the trends were similar among different varieties, indicating that pod formation took significantly longer under LD conditions compared to SD conditions (Figure 1 and Figure S1). By contrast, under the SD conditions, most of the first-opened flowers successfully initiated pod setting just about three days after R1 (Figure 1a–c, Figures S1c and S2a). These results indicate that the photoperiod affected the pod-setting time after flowering. Why does soybean require more time to initiate pod setting under LD conditions? We found that under LD conditions, the first-round opened flowers of W82 gradually fell off at most nodes, but later buds continued to be produced, and these second-round opened flowers gradually developed into pods. Approximately 16 days after R1, most buds fell off from the nodes on the main stem (Figure 1b and Figure S2b). This is one of the reasons for the longer time interval between flowering and pod setting under LD conditions. 

### 3.2. Soybean Remains Photoperiod-Sensitive After Flowering

**Figure 2:**
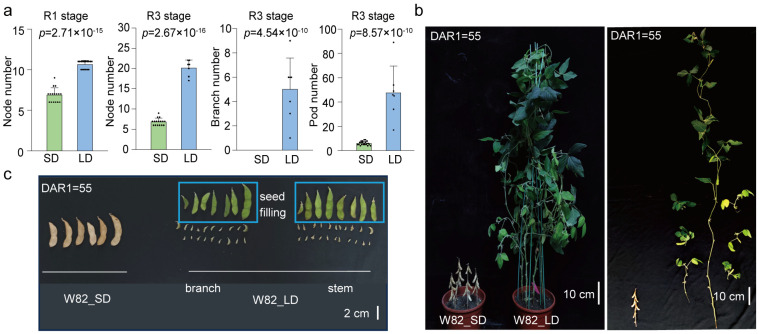
Differences in growth and architecture of soybean cultivar W82 under differing photoperiod conditions. (**a**) Numbers of nodes on the main stem at the flowering stage (R1) and pod-setting stage (R3), and pod and branch numbers at R3 of W82 under SD (12 h light/12 h dark) and LD (16 h light/8 h dark) conditions. (**b**) Phenotype of W82 under SD and LD photoperiod conditions. Seeds were sown at the same time, while plants matured faster, were shorter, produced fewer nodes, and had fewer branches under SD than LD. (**c**) Pod growth status of SD and LD conditions at 55 days after R1 (DAR1). When SD plants reached their maturity stage, the total pod number of per plant and developmental stages under two photoperiod conditions were observed at this timepoint. Brown pods are ripe, and green are unripe. Under SD conditions, plants had no branches. Pods on branches and the main stem under LD conditions were present here. All data are presented as means ± S.E.M. One-tailed, two-sample *t*-tests were used to generate the *p*-values.

Soybean is known to be sensitive to the photoperiod before flowering [5,36,48]; however, the post-flowering sensitivity and mechanisms remain unclear. We grew the soybean cultivar W82 under LD (16 h light/8 h dark) and SD (12 h light/12 h dark) conditions and investigated its phenotypes at R1, R3, and mature stages. W82 displayed different flowering times and plant architectures under different photoperiods. Under SD conditions, plants were smaller, with fewer nodes, branches, and pods (Figure 2a–c). During the period from flowering (R1) to post-flowering (R3), the plants under LD conditions gained about 10 nodes, while those under the SD conditions only gained 2 nodes during this period (Figure 2a). These observations indicate that the post-flowering photoperiod sensitivity not only affected the timing of pod initiation, but also affected plant architecture traits, such as the node number. Does the significant difference in pod formation rates between LD and SD conditions solely result from differences in plant architectures?

**Figure 3:**
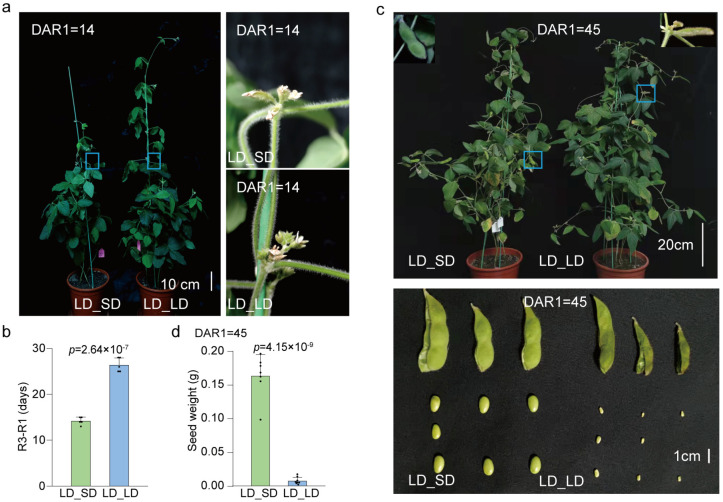
Soybean remains photoperiod-sensitive after flowering. In order to detect the effect of photoperiod transfer on post-flowering development, half of the 10 plants grown in LD (16 h light/8 h dark) conditions were transferred to SD (12 h light/12 h dark) conditions at the R1 stage (named the LD_SD group), while the remaining 5 plants continued to grow under continuous LD conditions (named LD_LD, the control group). (**a**) Pod setting was initiated about 14 days after transplantation in the LD_SD plants, but not in the LD_LD conditions. (**b**) The time required from R1 (time of the first opened flower) to R3 (initiation time of podding) of the LD_SD and LD_LD experiment groups. (**c**) Three representative pod and seed statuses 45 days after the photoperiod transfer treatment. (**d**) Fresh seed weight of LD_SD and LD_LD groups at 45 days after the photoperiod transfer treatment. All data are presented as means ± S.E.M. One-tailed, two-sample *t*-tests were used to generate the *p*-values.

**Figure 4:**
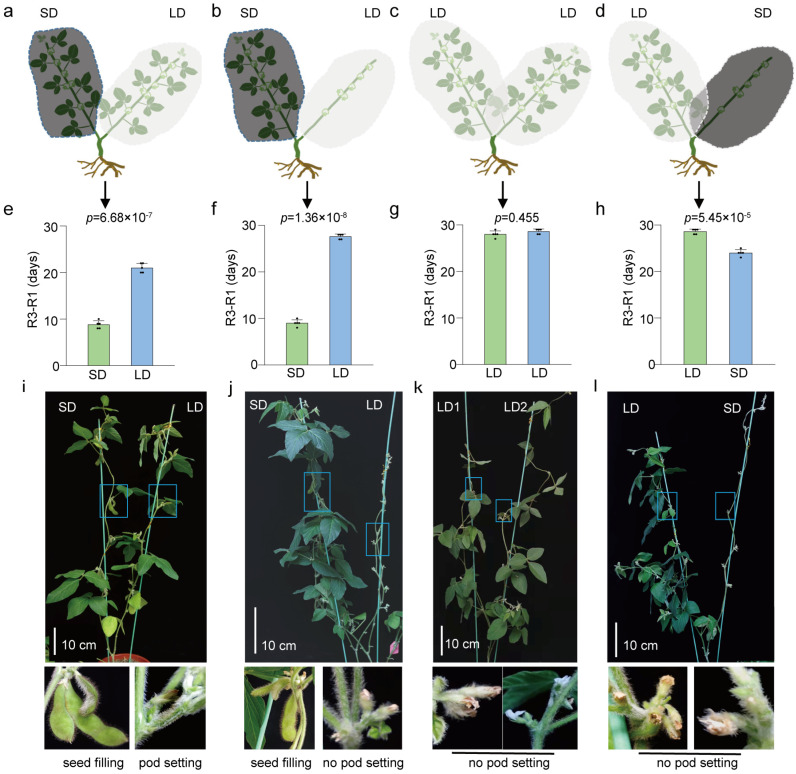
Branch-specific photoperiod treatments revealed that the leaves were responsible for the pod-setting signal. (**a**) Four groups of branch-specific photoperiod treatments. The shoot apical meristem (SAM) was removed from soybean seedlings in LD (16 h light/8 h dark) conditions, resulting in the simultaneous development of two lateral branches. Different photoperiod treatment combinations were applied to two branches after flowering (R1). (**a**) SD&LD group: Under normal long-day (LD; 16 h light/8 h dark) conditions, one branch was covered with a black plastic bag at ZT12 to simulate the short-day (SD; 12 h light/12 h dark) conditions, while another branch was treated with a transparent plastic bag to maintain the LD conditions. In order to demonstrate that the leaves are the main organs sensing the photoperiod and transmitting podding signals, the leaves were also removed from either the SD or LD branches undergoing the SD&LD treatment (**b**,**d**). (**c**) LD&LD group: As a control, both branches were covered with transparent plastic bags to maintain LD conditions. (**e**–**h**) Days required from flowering to podding (R3–R1) of four groups in (**a**–**d**). (**i**–**l**) The phenotypes of the different photoperiod combinations described in (**a**–**d**) at 15 days after treatment. Branches in the SD condition with leaves successfully set pods, and at 15 days the pods reached the seed-filling stage (**i**,**j**). Under the LD, the pod-setting time was later than that of the SD, whether or not the leaves were removed. All data are presented as means ± S.E.M. One-tailed, two-sample *t*-tests were used to generate the *p*-values.

To further observe post-flowering photoperiod sensitivity, we employed a photoperiod transfer experiment, and simulated LD (16 h light/8 h dark) and SD (12 h light/12 h dark) conditions on the two branches of the same decapitated soybean plant. In the photoperiod transfer experiment, the soybean plants of W82 were grown under LD (16 h light/8 h dark) conditions until the R1 stage, after which half were transferred into SD (12 h light/12 h dark) conditions (LD_SD group), with the remaining half continuing to grow under the LD conditions as a control group (LD_LD group). Compared to the LD_SD group, the LD_LD group took longer days to initiate podding (Figure 3). About 14 days after being moved to the SD conditions, the soybean plants of W82 began to successfully set pods, but there was no pod setting under continuing LD conditions (Figure 3a,b). At 45 days after the photoperiod transfer treatment, pod and seed development under SD conditions were significantly further than under LD conditions, indicating that SD conditions promoted faster development after flowering (Figure 3c). In the experiment of LD and SD simulation on the same plant, to obtain long branches at similar stages of growth, the SAMs of the soybean plants were removed five days after their emergence under LD conditions (Figure S3a,b). This released apical dominance, resulting in two symmetrical axillary buds that later developed into two long branches, unlike untreated soybean plants with a single main stem and short branches (Figure S3c). Next, different photoperiod treatment combinations were applied to the two long branches of each SAM-removed plant after flowering (R1 stage); in the SD&LD combination, one branch was covered with a black plastic bag at ZT12 (LD condition) to simulate the SD condition, while the other branch was covered with a transparent plastic bag to maintain the LD condition, with bags removed daily at ZT0 (Figure 4a). Under the SD&LD treatment, the branch under the simulated SD conditions set pods earlier than those under the LD conditions, with podding occurring approximately 9 days after shading treatment and reaching the filling stage 15 days after treatment. No pod formation occurred even after prolonged exposure to LD conditions (Figure 4e,i).

These results show that soybean remained sensitive to the photoperiod even after flowering, especially reflected in different pod-setting times, suggesting that plant architecture may not be the sole factor contributing to this difference.

### 3.3. The Photoperiod-Regulated Pod-Setting Signal Is Mainly Induced in and Transmitted Within the Leaves

How does the photoperiod affect the conversion of open flowers to pods or shedding? We set different photoperiodic conditions for branches on the same plant; in addition, to prove that leaves are the main organs for perceiving the photoperiod and transmitting podding signals, we removed the leaves of the branches under the different photoperiod conditions of the SD (12 h light/12 h dark) and LD (16 h light/8 h dark) treatments (Figure 4a–d). For this study, five-day-old soybean seedlings were decapitated at the cotyledon stage, and there were no leaves from other parts of soybean, except the two branches. Our treatments included four experimental groups: SD&SD and SD&LD (with no leaves after R1 under the LD condition), and LD&LD and LD&SD (with no leaves after R1 under the SD condition). The LD&LD combination was a control, in which both branches were covered with transparent plastic bags (Figure 4c). The pod-setting time under the SD conditions was prolonged by removing the leaves in the LD&SD (with no leaves after R1) group (Figure 4e,h). Under LD conditions, the pod-setting time was longer than that under SD conditions, regardless of leaf removal (Figure 4e–h). However, comparing the branches at stage R3 under LD conditions in the four groups, we found that the onset of pod formation in the LD&SD treatment group occurred approximately one week earlier (about 21 days) than in the other three groups (about 30 days; Figure 4e–h). The pod formation signal should be perceived by the leaves, transmitted downward, and communicated between different branches. Moreover, the signal inducing short-day pod formation was stronger than that promoting long-day flower abscission. From these results, we inferred that the leaves were the main light sensors, and that the photoperiod signal was mainly induced in the leaves, which then transmitted the signal to form pods to the flowers.

### 3.4. E1 Is Downstream of the EC in Controlling Pod-Setting Time

As reported, in the homologs of *PHYA*, members of the evening complex (*EC*), *E2* and *E1*, are the major genetic players in the control soybean photoperiod sensitivity, and their functions are mainly described in regulating the flowering time [5,36,48]. To further explore the genetic pathway underlying how the photoperiod affects the pod-setting time after flowering, we investigated the pod-setting time of photoperiod-insensitive mutants under different photoperiod conditions. The early-flowering triple-mutant *e1-3m*, which is insensitive to the photoperiod, underwent early pod setting after flowering, with no differences under different photoperiods (Figure S4a–c). The late-flowering double-mutant *lux-2m*, which was also insensitive to the photoperiod, had later flowering times and pod-setting times than the wild type under particularly long-day (20 h light/4 h dark) conditions (Figure S4d,e). To examine whether the difference in flowering and podding times between the wild-type and late-flowering mutants disappears under extremely long photoperiods, we selected exceptionally long photoperiods. *e1-3m* was crossed with *lux-2m* to obtain the *e1-3m lux-2m* quintuple mutant. Under LD (16 h light/8 h dark) conditions, *e1-3m lux-2m* showed early pod setting, which was similar to the *e1-3m* phenotype (Figure S4f). *Luxs* are parts of the *EC* in the circadian clock [4,5,49]. This indicates that *E1* is downstream of the *EC* in controlling the initiation of pod setting, and that pre- and post-flowering photoperiodic sensitivity may be controlled by the same genes. However, after the input of the photoperiodic signal, the response genes controlling different development processions may be different.

### 3.5. Photoperiod Affects Pistil Development

**Figure 5:**
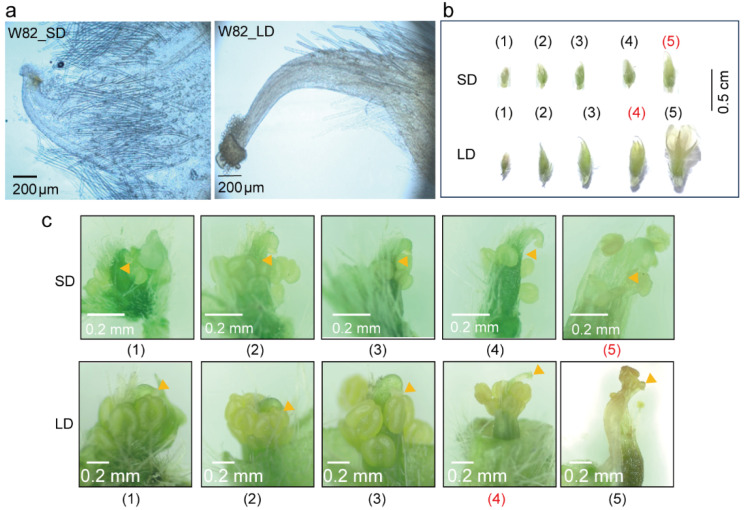
Photoperiod affects style morphology and the development of the pistil and stamen of soybean. (**a**) Phenotype of the styles of opened flowers of W82 under SD (12 h light/12 h dark) and LD (16 h light/8 h dark) conditions, respectively. (**b**) Flowers or buds in different development stages in an inflorescence under SD and LD conditions of W82. (**c**) Growth status of the pistil and stamen of the bud or flower in (**b**). The numbers marked in red represent the buds with pollen grains dispersed from anthers. The orange triangle represents the position of the stigma.

In previous experiments, we found that flowers opened in LD conditions tended to fall before pod formation. We sought to investigate whether there were differences in pollen viability and pistil morphology between LD and SD conditions, leading to differences in pod formation times. We collected pollen from W82 under LD and SD conditions and conducted pollen germination experiments in vitro. Results revealed no significant differences in the pollen tube length and pollen germination rate (Figure S5a–d). Additionally, unopened flower buds under LD and SD conditions were emasculated and artificially pollinated, and pollen tubes were able to germinate normally in vivo (Figure S5e,f). The effect of the photoperiod on pollen viability may be minimal. We found that there were morphological differences in pistil morphology under LD and SD conditions (Figure 5a). This morphological difference led to a similar height of the pistil and stamen when the stamen began to disperse powder under short-day conditions (Figure 5b,c and Figure S6a,c,d), facilitating rapid and successful pollination. The height of the stamen was lower than that of the pistil under long-day conditions (Figure 5b,c and Figure S6a,c,d), which was not conducive to rapid pollination. Flower buds or open flowers exhibited similar external sizes and shapes under both LD and SD conditions, but significant differences existed in stigma sizes (Figure S6a,b,e,g). The morphology of pistil styles varied greatly in the late development stage of buds. Under SD conditions, a hook-like structure was present at the apex of the stigma, whereas under LD conditions, the curved hook was less pronounced. When moved from LD to SD for a period of time, the hook structure at the apex of newly emerged flower buds became pronounced (Figure 5a–c and Figure S7).

**Figure 6:**
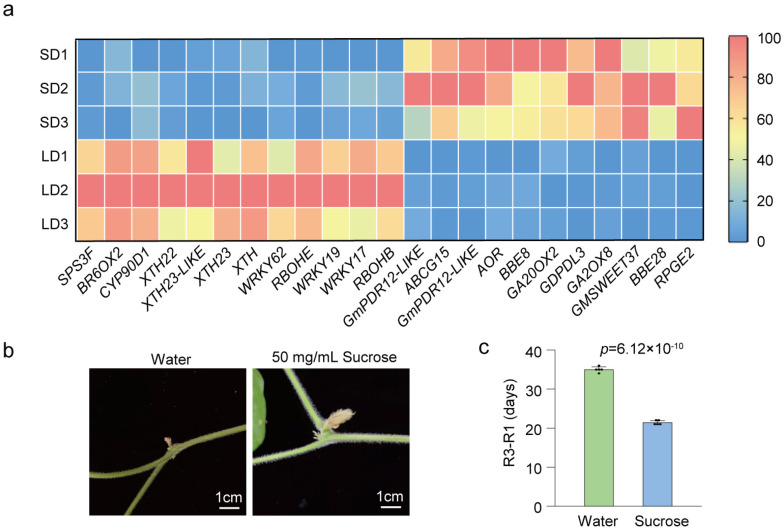
Comparison of transcript activities and plant hormone contents in pistils of SD (12 h light/12 h dark) and LD (16 h light/8 h dark) conditions. (**a**) Some differentially expressed genes in pistils of SD and LD conditions. (**b**) Pod or flower morphology at the fourth upmost node of control groups and the external application of 50 mg/mL sucrose solution groups. (**c**) External application of sucrose shortens the time required for initial pod setting under LD conditions. All data are presented as means ± S.E.M. One-tailed, two-sample *t*-tests were used to generate the *p*-values.

**Figure 7:**
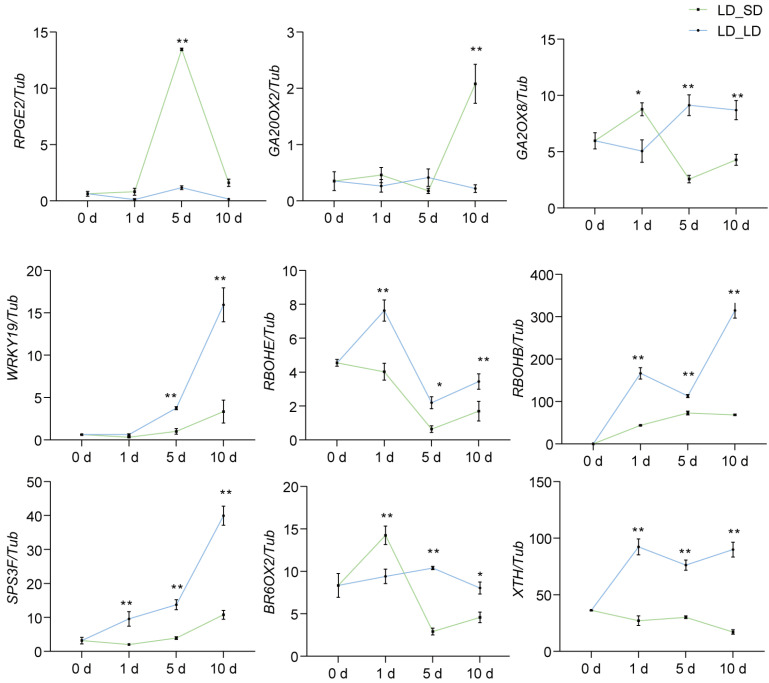
Relative expressions of pistil growth- and development-related genes in W82 under LD_LD and LD_SD conditions at different times. Relative expression of *RPGE2*, *GA20OX2*, *GA2OX8*, *WRKY19*, *RBOHE*, *RBOHB*, *SPS3F*, *BR6OX2*, and *XTH* in W82 plants under continuous long-day (16 h light/8 h dark) conditions (LD_LD) and the short-day (12 h light/12 h dark) transfer experimental group (LD_SD) after R1. We took the buds of the top-three nodes at day 0, day 1, day 5, and day 10 after R1, and isolated the pistil for analysis of these gene expression levels. Data are shown relative to the control gene *Tubulin* and represent means ± SD for three biological replicates. **: *p* < 0.01; *: *p* < 0.05.

**Figure 8:**
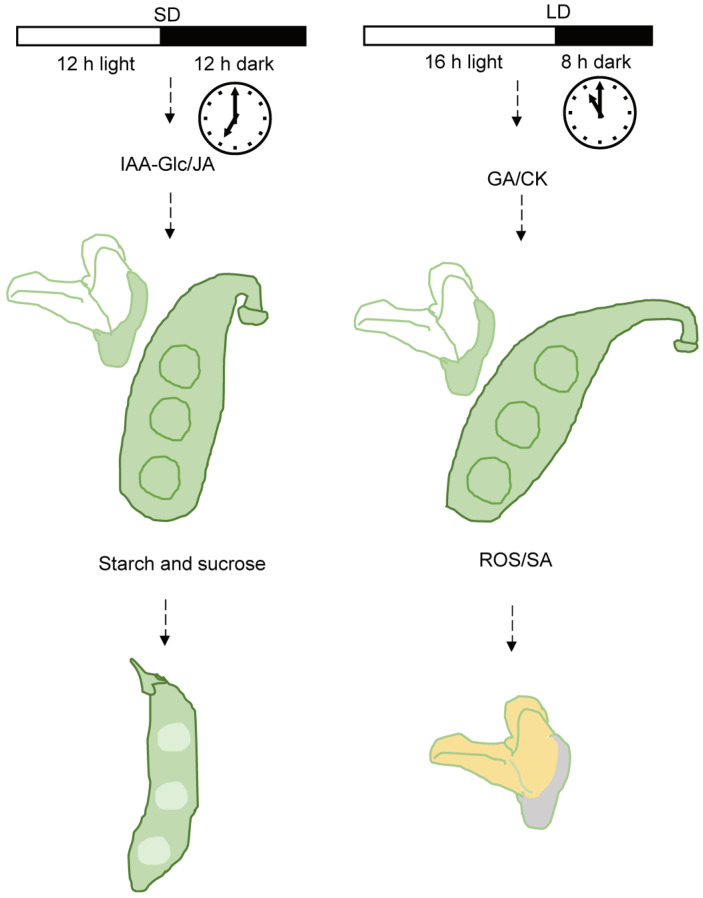
A proposed working model for SD (12 h light/12 h dark) and LD (16 h light/8 h dark) conditions to regulate soybean photoperiod podding. SD conditions affect the concentration of auxin on one side of the style, leading to polarized growth of the style and the formation of a curved hook at the top. The hook makes stigma easy to contact with pollen emitted by the stamens. In addition, a higher concentration of jasmonic acid facilitates fertilization of the pistils. Photosynthetic metabolites are transported from the source to the sink (flower), facilitating pod formation. Under LD conditions, hormone levels are different from those under SD conditions. The concentration of gibberellins and cytokinins in the style is relatively high, leading to sustained elongation of the style, which is not beneficial to contact with stamens and fertilization. In addition, genes related to the respiratory burst oxidase homologue (*RBOH*) are highly expressed and might lead to a high content of reactive oxygen species in flowers, and the level of salicylic acid hormone is high, resulting in flower abscission and not conducive to rapid pod formation.

Which genes and plant hormones affect pistil development under different photoperiod conditions? We collected flower buds under LD and SD conditions, measured plant hormone levels, and isolated pistils for RNA extraction, constructing RNA-Seq libraries and analyzing differentially expressed genes. Simultaneously, we analyzed the relative expression levels of differentially expressed genes in the buds of the top-three nodes of soybean plants under the continuous long-day (LD_LD) and LD_SD groups at R1, 1 day, 5 days, and 10 days after the R1 stage. According to the sequencing results (Supplementary Table S1), the regulatory pathways of differentially expressed genes involved the MAPK signaling pathway, starch and sucrose metabolism, photosynthesis, and plant hormone signal transduction (Figure S8). We identified at least 23 DEGs that might affect soybean pod formation (Figure 6a; Supplementary Table S2). We selected 9 genes from the 23 DEGs for PCR verification. Consistent with our qRT-PCR analysis (Figure 7), *REPRESSOR OF PHOTOSYNTHETIC GENES 2* (*RPGE2*), *GIBBERELLIN OXIDASE 8* (*GA2OX8*), and *GA2OX2* were upregulated upon transfer to SD conditions, while *WRKY19*, *RESPIRATORY BURST OXIDASE HOMOLOGUE E* (*RBOHE*), *RBOHB*, *SUCROSE PHOSPHATE SYNTHASE 3F* (*SPS3F*), and *Xyloglucan Endotransglucosylase/hydrolases* (*XTHs*) were strongly inhibited (Figure 6a and Figure 7). Studies have indicated that in this research, by determining the contents of eight major types of phytohormones in flower buds, as expected, the content of some plant hormones varied in the buds under LD and SD conditions (Figure S9; Supplementary Table S3). The linear equations of the standard curve, along with the correlation coefficients for the detected substances in this study, are presented in Supplementary Table S5. The contents of gibberellin 1, 3, 7 (GA1, GA3, and GA7), and salicylic acid were higher under LD conditions. The contents of auxin and jasmonic acid were higher under SD conditions. It was discovered that the content of active cytokinins (N6-isopentenyl-adenine-9-glucoside (ip6G), dihydrozeatin-7-glucoside (DHZ7G), and dihydrozeatin ribonucleoside (DHZR)) in flower buds under short-day conditions was significantly higher than that under long-day conditions. The content of cis-Zeatin riboside (cZR) was significantly higher in long-day conditions (Figure S9). Under LD conditions, after flowering (R1 stage), a 50 mg/mL sucrose solution was applied on the leaves, and the control group was sprayed with the same amount of water. The results showed that the external application of sucrose solution could promote pod formation (Figure 6b,c). All these results suggest that the photoperiod may control soybean pod formation and development by regulating multiple gene pathways and plant hormones (Figure 8).

## 4. Discussion

The mechanisms of photoperiod perception before and after flowering are similar. The photoperiod regulates various growth and development processes, such as floral induction, stem termination, and pod development, in the post-flowering reproductive growth stage [18,24,50]. Previous studies found that exposing soybean plants to long-day conditions during post-flowering reproductive growth stages extended the R3–R6 period, with seed development and seed number positively correlating with the duration of the R3–R6 stage [20]. These results indicated that soybean plants remain sensitive to the photoperiod during the post-flowering R3-R6 stages. In this study, we found that different soybean cultivars were sensitive to the photoperiod during the initiation of podding after flowering in laboratory-controlled conditions. SD conditions promoted pod formation, while LD prolonged the duration of the R1 to R3 stage. The photoperiod-insensitive mutants used in this study might provide a basis for further research on the mechanism of photoperiod-sensitive-related genes in regulating the pod initiation time. The photoperiod-insensitive *lux-dm* and *e1-3m* mutants [5,36] displayed two extreme phenotypes. The *lux-dm* mutants had a late flowering time and produced more stem nodes, branches, and leaves than wild-type soybean plants [5,36], while the *e1-3m* mutant had a smaller morphology, with few nodes and an early flowering time [36]. In this study, we found that *e1-3m* had a short R1–R3 stage (about 5 days) under both LD and SD conditions, and the pod initiation time was not sensitive to the photoperiod. The *lux-dm* mutant exhibited a longer R1-R3 duration. Meanwhile, the *e1 e1la e1lb lux1 lux2* quintuple mutant showed an R1–R3 duration similar to *e1-3m*, suggesting that *E1* and *E1-like* function downstream of the *EC* in controlling the pod-setting time, with *EC* being entirely dependent on *E1*. The mechanisms of photoperiod signal sensing and transmission may remain conserved before and after flowering. *E1*, *E1-like*, and *EC* have been reported to play major roles in floral induction [5,11,36], but their roles in post-flowering reproductive development remain undetermined. Increasing research attention is being paid to the effects of growth period genes on post-flowering development [51,52].

### Photoperiod Affects the Internal Environment of the Flower to Influence Pod Formation

The coordination of flower development and fertility is regulated by endogenous developmental signals, such as the phytohormones jasmonates (JAs), auxin, and gibberellin, as well as environmental cues [53]. We found that under LD photoperiod conditions, the first-opened flowers typically dropped, and the second-round flowers slowly turned into pods. In our study, we found that the pistil style of W82 exhibited different morphologies: when the anther of the stamen was dispersed, the stigma was higher than that of the stamen. Under SD conditions, there were apical hook formations in a flower-style-like hook in emerging seedlings. The longer style length in rice influenced the stigma exertion and increased the outcross rate of the male sterile line and the yield of hybrid F1 seeds. The elongation of the cell length in the style was associated with a higher GA4 content in the pistil [54]. We found that under LD conditions, endogenous GA1, GA3, and GA7 contents in flower buds were higher than that under SD conditions, but IAA-Glc and JA-Ile contents were lower. Apical hook formation involves a gravity-induced auxin maximum on the eventual concave side of the hook [55]. Jasmonoyl-L-isoleucine (JA-Ile) is a biologically active form of JA. JA-deficient mutants exhibited low fertilization rates and abnormal flower formation [56,57,58,59,60]. The *jasmonic-acid-insensitive 1-1* (*jai1-1*) mutants in tomato exhibited arrested flower bud development just before flower opening by abolishing the peaks of JA biosynthesis, and *SlMYB21* expression in flower buds within ~2 days before flower opening [61,62]. These results suggested that JA plays a crucial role in flower development and fertility in rice and tomato. These findings provide compelling evidence for the pivotal role of cytokinin in regulating the growth of reproductive meristems and organs [63,64]. The mutant of cytokinin oxidase/dehydrogenase enzyme 3 and 5 genes (*ckx 3 ckx5*) in *Arabidopsis thaliana* had a higher concentration of the biologically active t trans-zeatin-type cytokinins and some of the iP-type cytokinins [63]. In oilseed rape (*Brassica napus*), the *Bnckx 3 ckx5* mutant had larger pistils, and the stigma of the pistil changed from its original vertical state to a slightly curved hook [64]. In our study, the variations in cytokinin levels in LD and SD flower buds may have contributed to the observed differences in pistil morphology. We performed RNA-seq on pistils of W82 flower buds under LD and SD conditions. Compared to pistils under LD conditions, plant cell wall remodeling enzymes *XTH22*, *XTH23*, and *XTH23-like* genes were significantly decreased under SD conditions (Figure 6a and Figure S9). *XTH22* and *XTH23* are known to play a role in cell elongation during flower development [65]. *RbohB* and *RbohE* genes were upregulated in LD conditions. Upon transition from LD to SD, their relative expression levels were downregulated (Figure 6a and Figure 7). *RBOH*s are reported to be crucial for ROS generation and are essential for precise flower and fruit abscission [66,67]. Previous studies have shown that under a photoperiod of approximately 14.5 h of light per day, about 21–28% of flowers and pods were aborted, which increased to 42–49% with shading treatments [31]. Top bud removal at each node, leaving only one remaining top bud, can reduce flower abscission rates, while shading treatments do not increase flower abscission rates [31]. This suggests that light/shade conditions are not directly responsible for flower/pod abscission signals; rather, a lack of nutrient supply leads to increased flower and pod abscission rates [31]. In our study, we found that even under SD conditions, which promoted pod setting, pod formation could not be achieved as rapidly after leaf removal as it was in the experimental group that retained its leaves (Figure 4d,h,i), likely because photosynthesis and assimilate accumulations were decreased. Enhanced carbon assimilation could reduce flower and pod abortion, as well as accelerating leaf expansion, seed yield, and the production of tuberous storage organs or fibers in various crops [68,69,70]. In this study, KEGG enrichment analysis of the DEGs in the buds before opening of soybean revealed that genes related to starch and sucrose metabolism and carbohydrate or energy metabolism were repressed under LD conditions. Application of an exogenous sucrose solution promoted pod formation. It was reported that during the early stages of seed development, embryos grew rapidly and acquired a large amount of sugar from liquid endosperm. An insufficient supply of nutrients from the endosperm to the embryo resulted in severe seed abortion and yield reduction [71]. Soybean seed development responded to the photoperiod, where the Dt1 protein physically interacted with the sucrose transporter GmSWEET10a, negatively regulating the transport of sucrose from the seed coat to the embryo, thus modulating the seed weight under LD conditions. *Dt1* exhibited pleiotropy in regulating both the seed size and stem growth habit in soybeans [72]. The photoperiod-insensitive mutants used in the present study might provide a basis for further studies into the mechanism by which the photoperiod-sensitive flowering pathway genes regulate the pod initiation time and pod number through the photoperiod-dependent regulation of the balance between source and sink tissues.

## 5. Conclusions

In this study, we found that soybean was still sensitive to the photoperiod after flowering, which was reflected in the effect of different pod-setting initiation times under long-day and short-day conditions. Future work should examine why different photoperiod-insensitive materials have different podding times. We will further examine the possibility and mechanisms of using photoperiod sensitivity in the post-flowering stage as a criterion for increasing yields by increasing seed numbers.

## Data Availability

Data supporting the findings of this study are available in the Supplementary Materials of this article.

## Supplementary Materials

The following supporting information can be downloaded at: https://www.mdpi.com/article/10.3390/biology13110868/s1. Figure S1: The difference in pod initiation times after flowering of Williams 82 (W82) under long-day (LD) and short-day (SD) conditions in a greenhouse. Figure S2: The growth and development process of soybean under different photoperiod conditions. Figure S3: Decapitation treatment of soybean plant. Figure S4: The pod initiation time of the triple mutant (*e1/e1la/e1lb*, *e1-3m*) is insensitive to the photoperiod. Figure S5: Effect of the photoperiod on pollen germination. Figure S6: The photoperiod affects pistil and stamen growth. Figure S7: The photoperiod influences the morphology of flower style. Figure S8: Enrichment analysis of differentially expressed genes in the pistil of W82 under LD (16 h light/8 h dark) and SD (12 h light/12 h dark) conditions. Figure S9: Some plant hormones with significant differences in content of SD and LD conditions. Table S1: A total of 5239 DEGs in pistils of Williams 82 between long-day and short-day conditions. Table S2: The 23 genes of the 5239 DEGs of Williams 82 between long-day and short-day conditions. Table S3: Phytohormones’ contents in flower buds under long-day and short-day conditions. Table S4: Primers for quantitative RT-PCR. Table S5: The linear equation for standard curves.
