# Supplementary material for: Post-Flowering Photoperiod Sensitivity of Soybean in Pod-Setting Responses
Source: Biology (Basel). 2024 Oct 25;13(11):868. doi: 10.3390/biology13110868 (PMC11592272; doi:10.3390/biology13110868)
Supplement: Supplementary file 1 [file biology-13-00868-s001.zip › Supplementary figures-revised2024.10.23.pptx]

## Slide 1
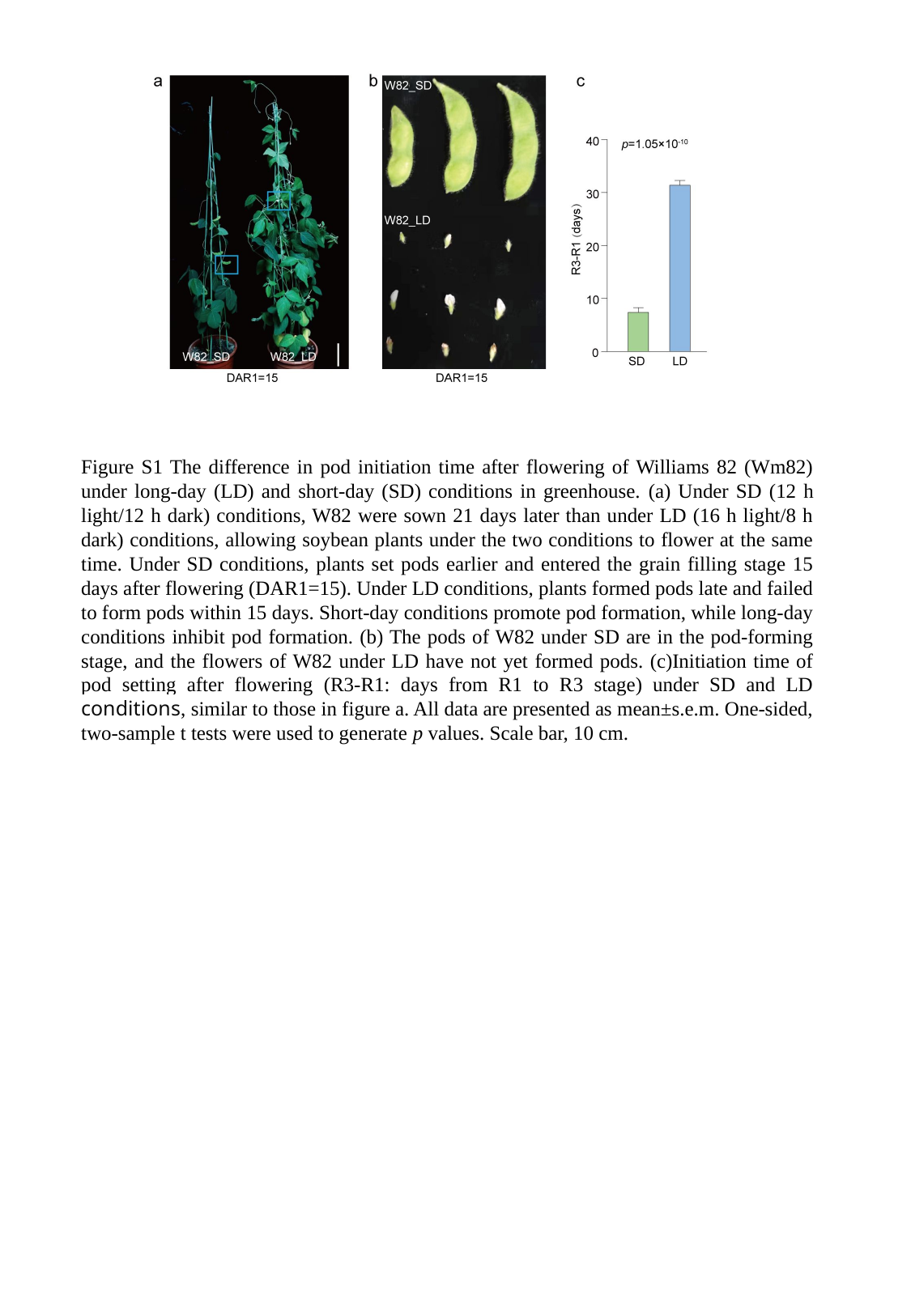

Figure S1 The difference in pod initiation time after flowering of Williams 82 (Wm82) under long-day (LD) and short-day (SD) conditions in greenhouse. (a) Under SD (12 h light/12 h dark) conditions, W82 were sown 21 days later than under LD (16 h light/8 h dark) conditions, allowing soybean plants under the two conditions to flower at the same time. Under SD conditions, plants set pods earlier and entered the grain filling stage 15 days after flowering (DAR1=15). Under LD conditions, plants formed pods late and failed to form pods within 15 days. Short-day conditions promote pod formation, while long-day conditions inhibit pod formation. (b) The pods of W82 under SD are in the pod-forming stage, and the flowers of W82 under LD have not yet formed pods. (c)Initiation time of pod setting after flowering (R3-R1: days from R1 to R3 stage) under SD and LD conditions, similar to those in figure a. All data are presented as mean±s.e.m. One-sided, two-sample t tests were used to generate p values. Scale bar, 10 cm.

## Slide 2
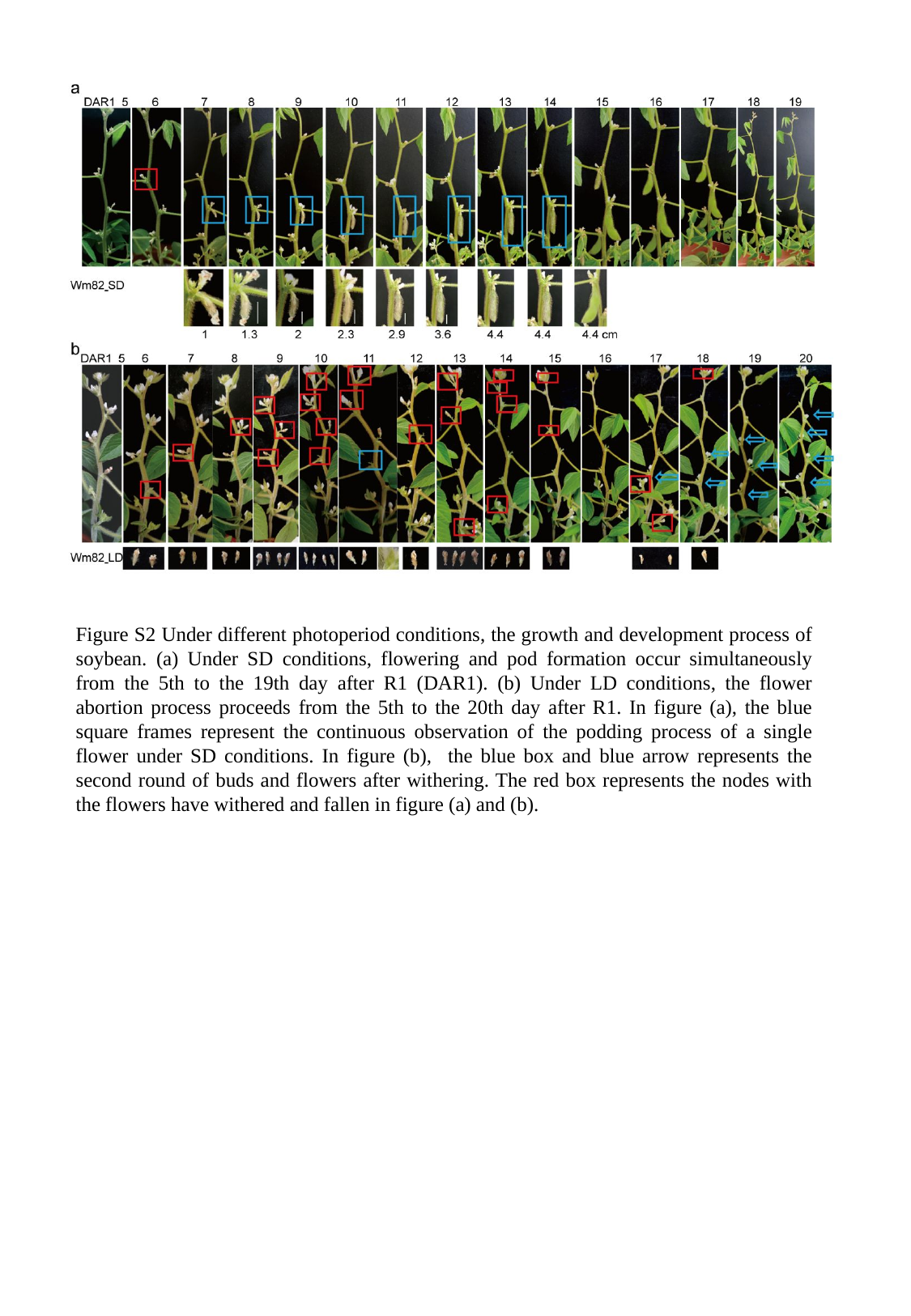

Figure S2 Under different photoperiod conditions, the growth and development process of soybean. (a) Under SD conditions, flowering and pod formation occur simultaneously from the 5th to the 19th day after R1 (DAR1). (b) Under LD conditions, the flower abortion process proceeds from the 5th to the 20th day after R1. In figure (a), the blue square frames represent the continuous observation of the podding process of a single flower under SD conditions. In figure (b), the blue box and blue arrow represents the second round of buds and flowers after withering. The red box represents the nodes with the flowers have withered and fallen in figure (a) and (b).

## Slide 3
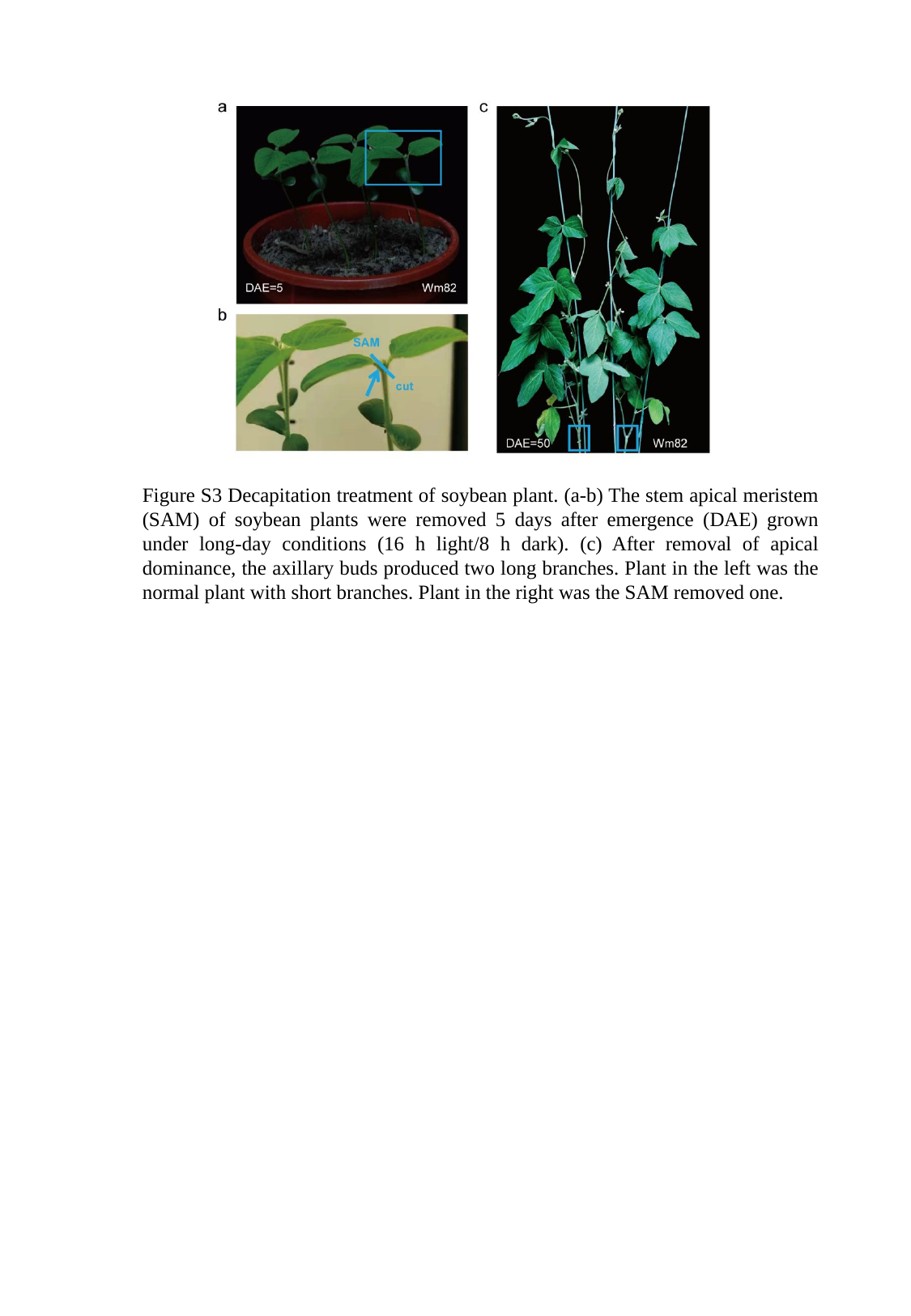

Figure S3 Decapitation treatment of soybean plant. (a-b) The stem apical meristem (SAM) of soybean plants were removed 5 days after emergence (DAE) grown under long-day conditions (16 h light/8 h dark). (c) After removal of apical dominance, the axillary buds produced two long branches. Plant in the left was the normal plant with short branches. Plant in the right was the SAM removed one.

## Slide 4
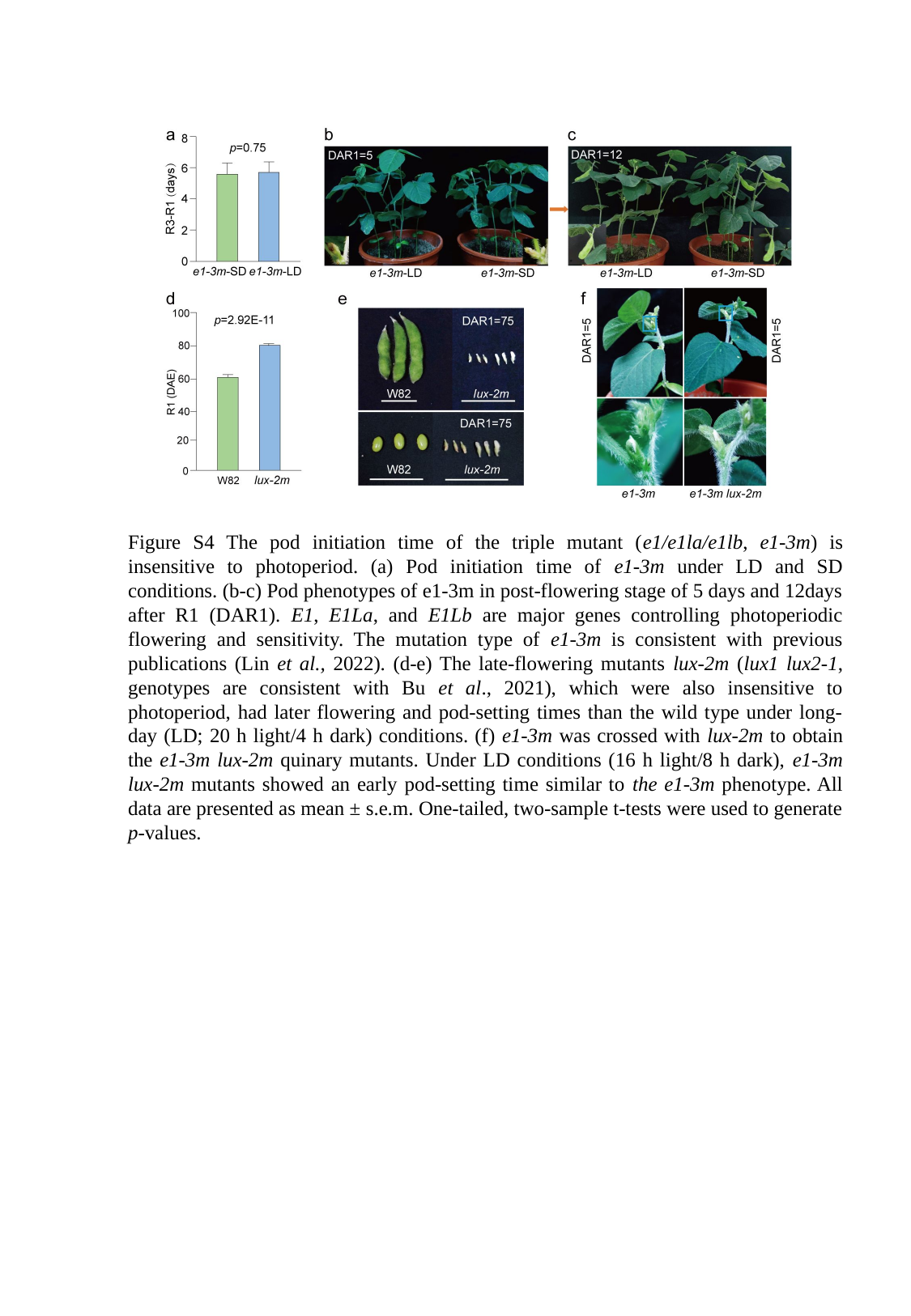

Figure S4 The pod initiation time of the triple mutant (e1/e1la/e1lb, e1-3m) is insensitive to photoperiod. (a) Pod initiation time of e1-3m under LD and SD conditions. (b-c) Pod phenotypes of e1-3m in post-flowering stage of 5 days and 12days after R1 (DAR1). E1, E1La, and E1Lb are major genes controlling photoperiodic flowering and sensitivity. The mutation type of e1-3m is consistent with previous publications (Lin et al., 2022). (d-e) The late-flowering mutants lux-2m (lux1 lux2-1, genotypes are consistent with Bu et al., 2021), which were also insensitive to photoperiod, had later flowering and pod-setting times than the wild type under long-day (LD; 20 h light/4 h dark) conditions. (f) e1-3m was crossed with lux-2m to obtain the e1-3m lux-2m quinary mutants. Under LD conditions (16 h light/8 h dark), e1-3m lux-2m mutants showed an early pod-setting time similar to the e1-3m phenotype. All data are presented as mean ± s.e.m. One-tailed, two-sample t-tests were used to generate p-values.

## Slide 5
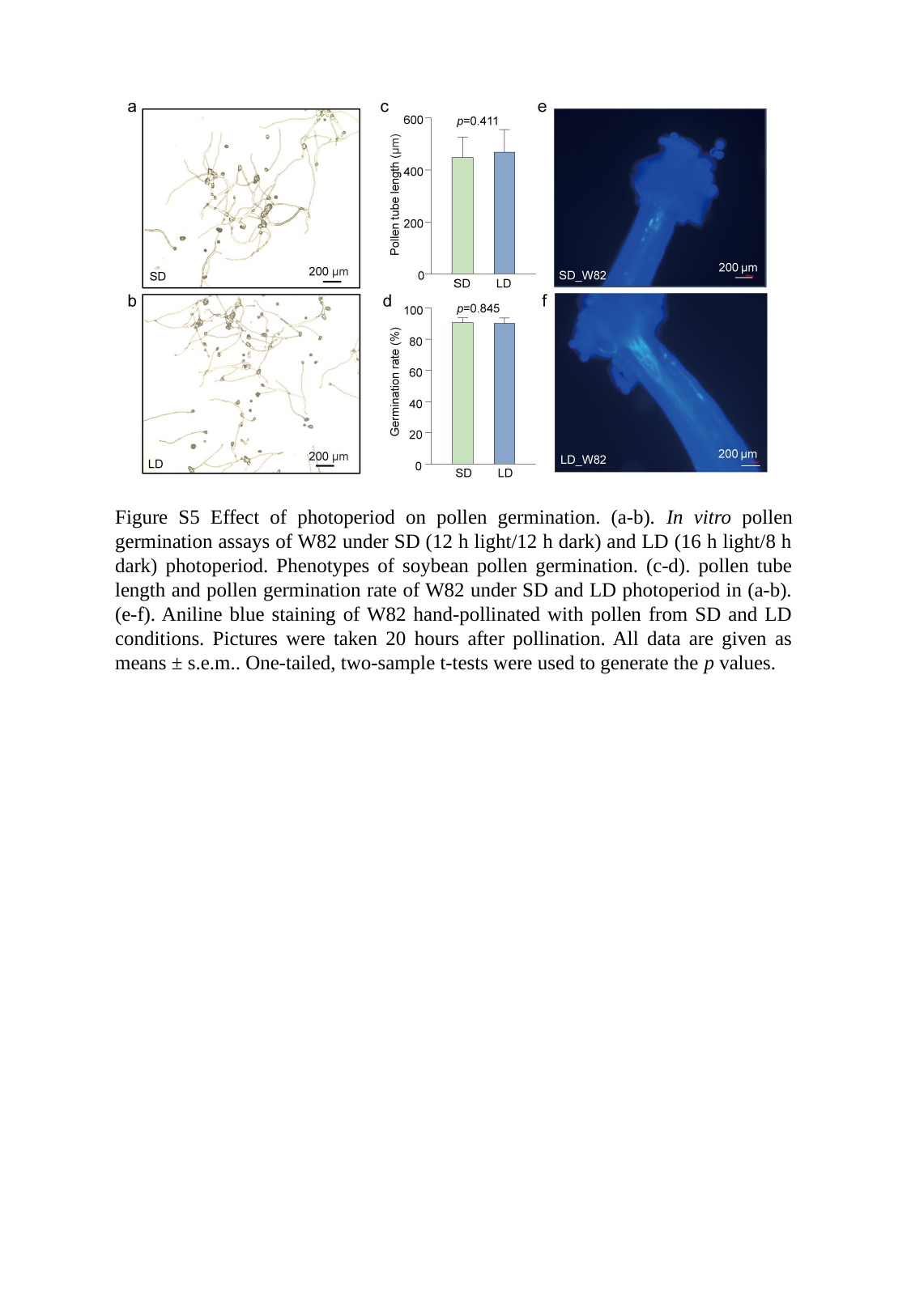

Figure S5 Effect of photoperiod on pollen germination. (a-b). In vitro pollen germination assays of W82 under SD (12 h light/12 h dark) and LD (16 h light/8 h dark) photoperiod. Phenotypes of soybean pollen germination. (c-d). pollen tube length and pollen germination rate of W82 under SD and LD photoperiod in (a-b). (e-f). Aniline blue staining of W82 hand-pollinated with pollen from SD and LD conditions. Pictures were taken 20 hours after pollination. All data are given as means ± s.e.m.. One-tailed, two-sample t-tests were used to generate the p values.

## Slide 6
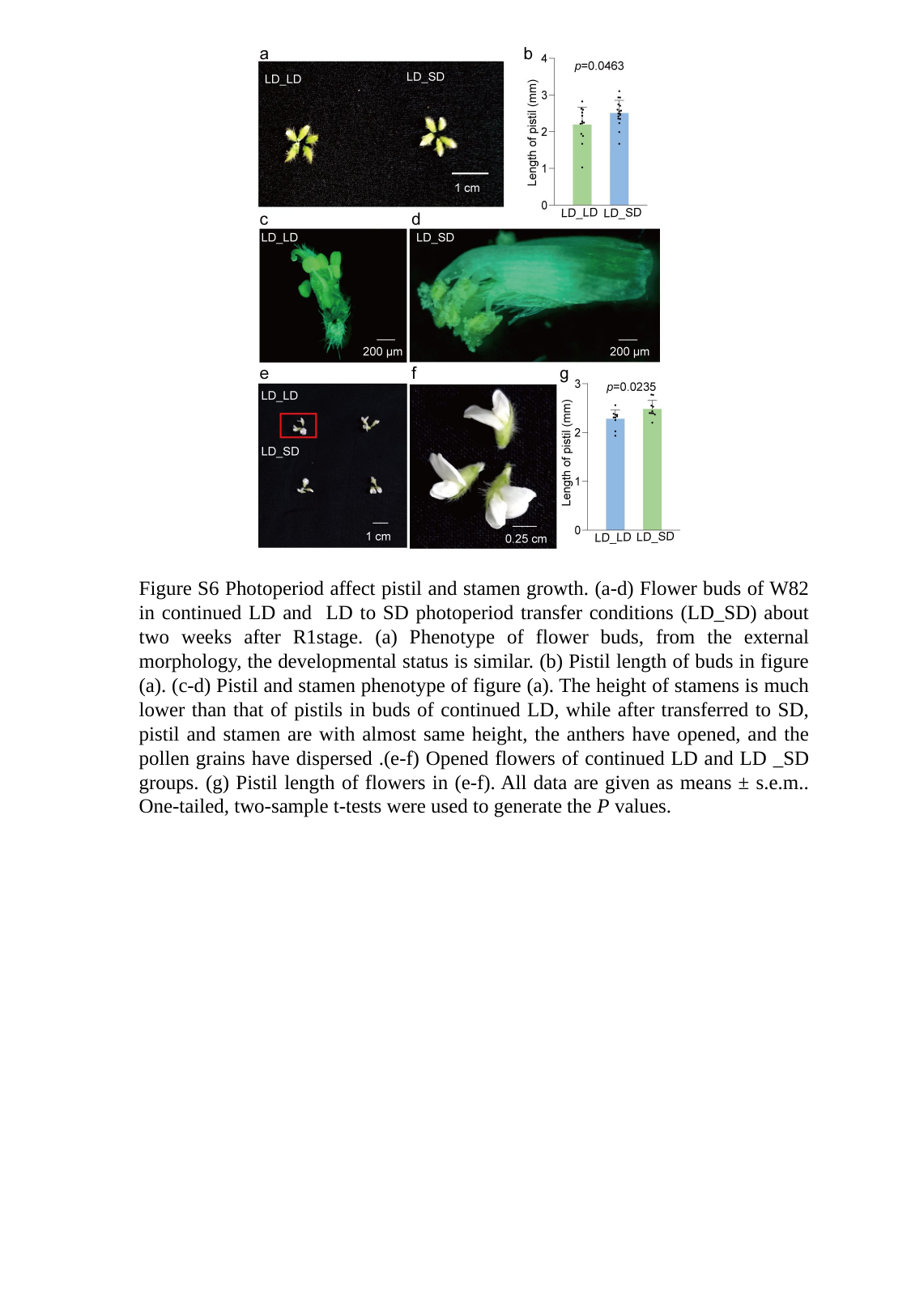

Figure S6 Photoperiod affect pistil and stamen growth. (a-d) Flower buds of W82 in continued LD and LD to SD photoperiod transfer conditions (LD_SD) about two weeks after R1stage. (a) Phenotype of flower buds, from the external morphology, the developmental status is similar. (b) Pistil length of buds in figure (a). (c-d) Pistil and stamen phenotype of figure (a). The height of stamens is much lower than that of pistils in buds of continued LD, while after transferred to SD, pistil and stamen are with almost same height, the anthers have opened, and the pollen grains have dispersed .(e-f) Opened flowers of continued LD and LD _SD groups. (g) Pistil length of flowers in (e-f). All data are given as means ± s.e.m.. One-tailed, two-sample t-tests were used to generate the P values.

## Slide 7
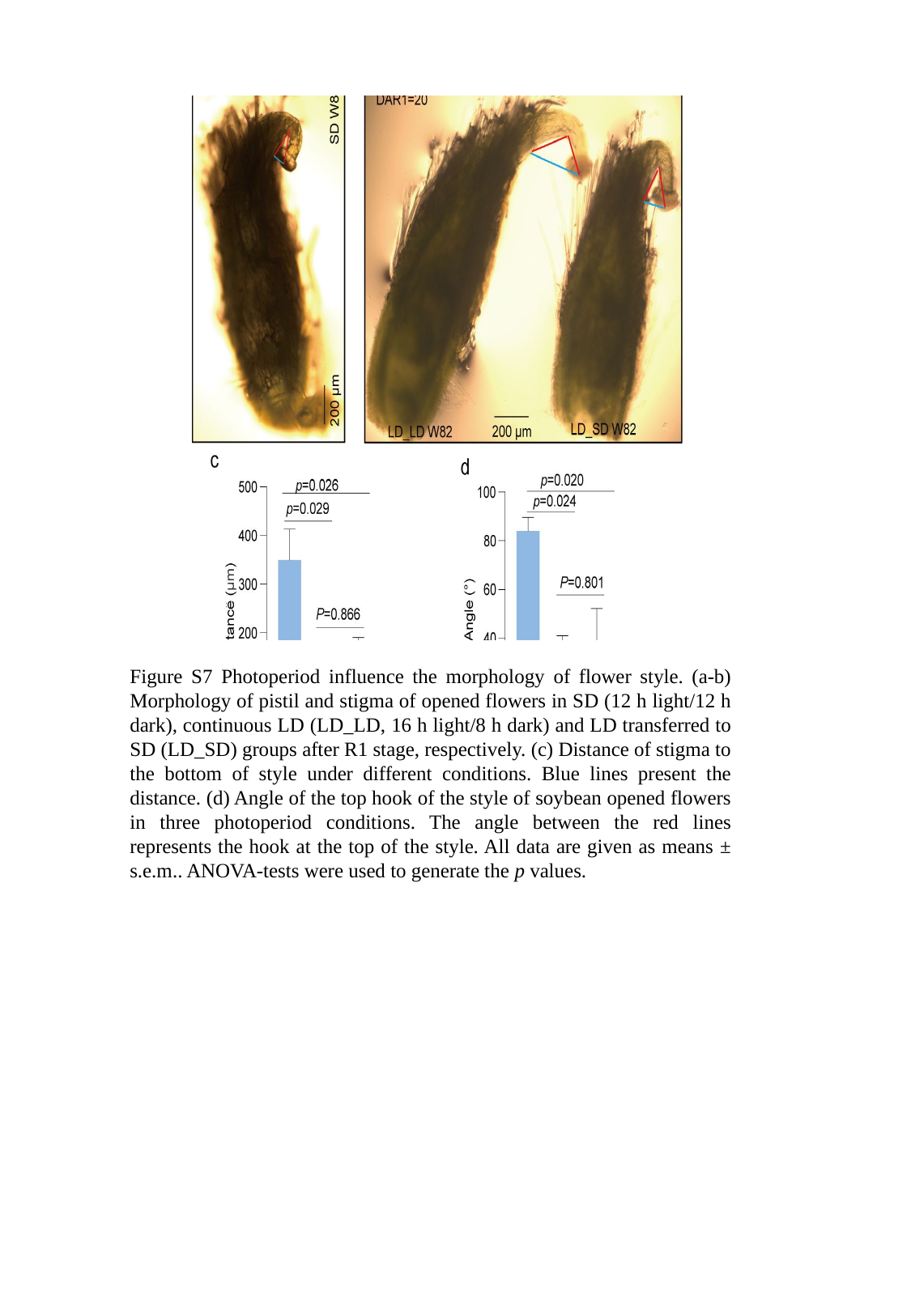

Figure S7 Photoperiod influence the morphology of flower style. (a-b) Morphology of pistil and stigma of opened flowers in SD (12 h light/12 h dark), continuous LD (LD_LD, 16 h light/8 h dark) and LD transferred to SD (LD_SD) groups after R1 stage, respectively. (c) Distance of stigma to the bottom of style under different conditions. Blue lines present the distance. (d) Angle of the top hook of the style of soybean opened flowers in three photoperiod conditions. The angle between the red lines represents the hook at the top of the style. All data are given as means ± s.e.m.. ANOVA-tests were used to generate the p values.

## Slide 8
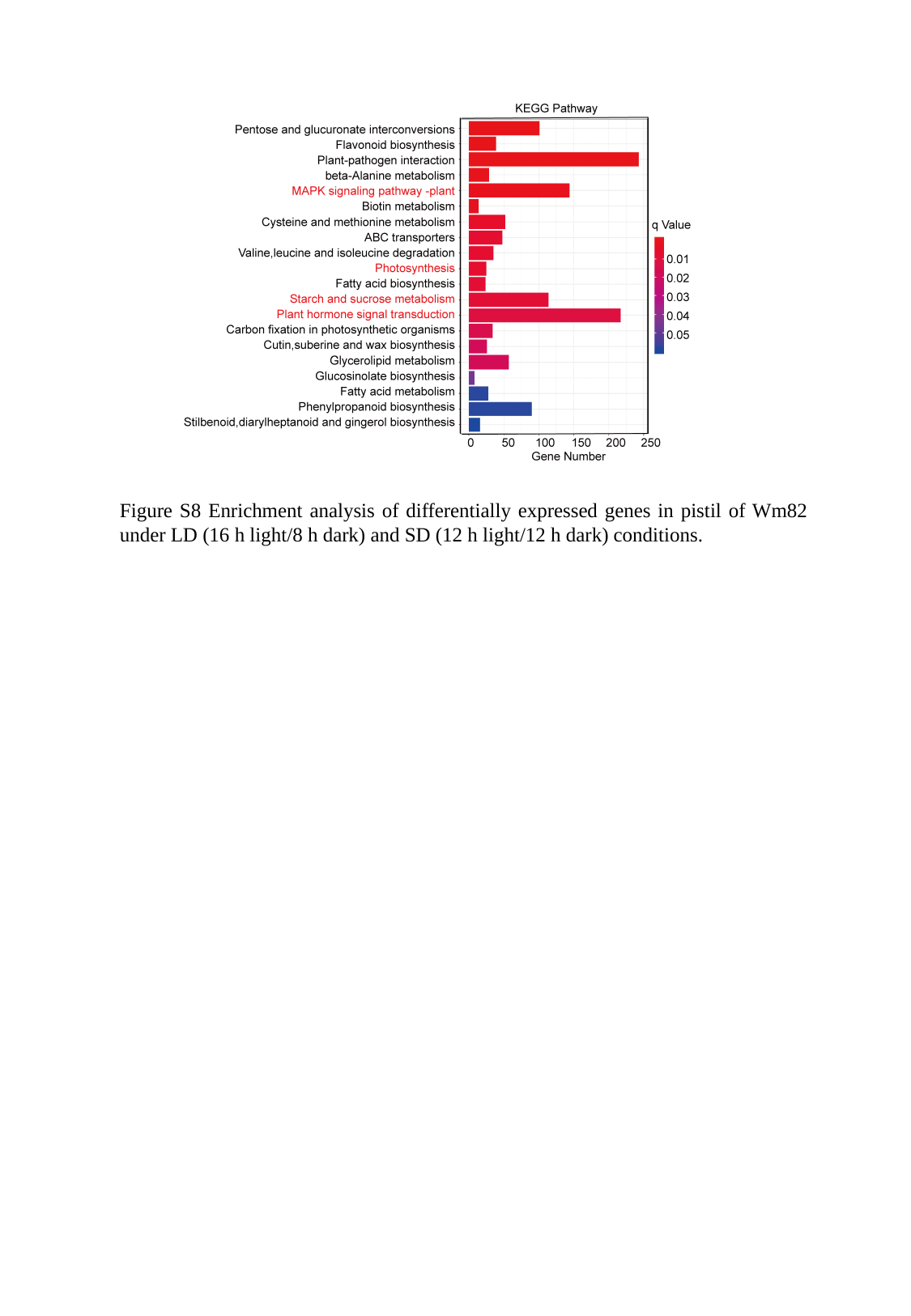

Figure S8 Enrichment analysis of differentially expressed genes in pistil of Wm82 under LD (16 h light/8 h dark) and SD (12 h light/12 h dark) conditions.

## Slide 9
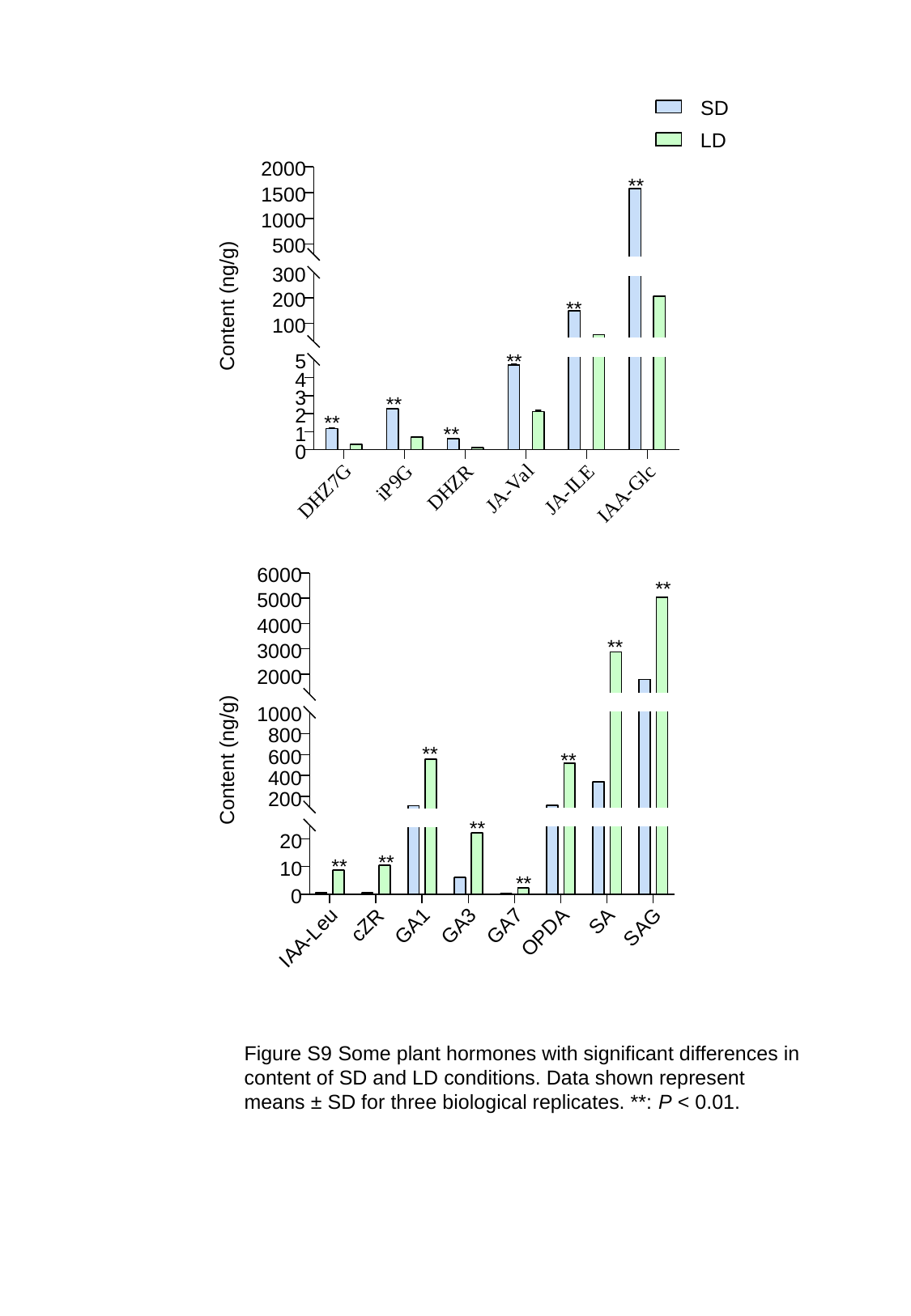

SD
LD
2000
**
1500
1000
Content (ng/g)
500
300
200
**
100
5
**
4
3
**
2
**
1
**
0
l
c
E
G
R
G
a
l
L
7
9
Z
V
G
I
Z
P
H
-
-
-
i
A
A
H
A
D
J
J
A
D
I
6000
**
5000
4000
**
3000
2000
Content (ng/g)
1000
800
**
600
**
400
200
**
20
**
**
10
**
0
u
1
3
7
A
A
R
G
e
A
A
A
S
Z
D
A
L
c
G
G
G
P
S
-
A
O
A
I
Figure S9 Some plant hormones with significant differences in content of SD and LD conditions. Data shown represent means ± SD for three biological replicates. **: P < 0.01.
